# Rapidly Developing Cataract in Young Adult Patients: Always a Matter for Further Evaluation

**DOI:** 10.7759/cureus.17312

**Published:** 2021-08-19

**Authors:** Naresh K Midha, Mahendra Kumar Garg, Deepak Kumar, Durga Shankar Meena, Gopal K Bohra

**Affiliations:** 1 Medicine, All India Institute of Medical Sciences, Jodhpur, IND

**Keywords:** hypoparathyroidism, cataract, intracranial calcification

## Abstract

A cataract in the young age group is uncommon and it is usually secondary to eye trauma, intraocular inflammation, uncontrolled diabetes mellitus, and hypoparathyroidism. We report a case of a rapidly developing cataract over two years in a 21-year-old female with extensive intracranial calcification due to primary hypoparathyroidism.

Chronic hypocalcemia due to underlying hypoparathyroidism results in cataract. Extensive bilateral intracranial calcification involving basal ganglia and white matter has been rarely reported in the literature. It occurs due to the chronic deposition of calcium-phosphorus complexes.

We would like to highlight that cataract in young patients is always a matter for further evaluation. Clinicians and ophthalmologists should be aware of hypoparathyroidism as a cause of bilateral cataracts. Early diagnosis of primary hypoparathyroidism can save patients from many complications.

## Introduction

Cataract is the main cause of blindness in developing countries. Cataract in the young adult age group is less common and it is usually secondary to eye trauma, intraocular inflammation, diabetes mellitus, inborn errors of metabolism, chronic hypocalcemia, and prolonged use of steroids. High myopia, prolonged dehydration, allergic dermatitis, and chronic uveitis are also mentioned risk factors for the development of cataract at a young age [1].

Young adult cataract due to hypocalcemia with underlying primary hypoparathyroidism is a rare condition. Hypocalcemia is characterized by muscle cramps, tetany, fatigue, paraesthesia, headache, and abdominal pain. Seizures and cardiac arrhythmias are life-threatening emergencies associated with hypocalcemia [2].

Ophthalmic involvements in hypoparathyroidism are well established and include papilledema and early cataract. Cataracts associated with primary hypoparathyroidism are mostly cortical and develop slowly while in secondary hypoparathyroidism, posterior subscapular cataract is more common, and it evolves rapidly [3].

We report a case of rapidly developing cataract over two years in a 21-year-old female due to primary hypoparathyroidism. Awareness of this rare complication can help in early diagnosis and further evaluation of cataract at the young age group

## Case presentation

A 21-year-old unmarried female presented to our outpatient department with complaints of muscular spasms from six years and difficulty in the vision of the right eye for the last year. The muscular spasms usually started from the legs and progressed to all extremities, worsened by cold. On further evaluation, she reported complaints of paraesthesia (tingling and pricking sensations) in limbs, around the mouth and difficulty in speech. The patient reported episodes of vertigo with a single episode of fall. She had no complaints of seizures in the past with no complaints of extrapyramidal symptoms.

The patient had a history of cataract surgery in the left eye two years ago. There was no history of comorbidities and any neck surgery like thyroidectomy. She denied any arthralgia, photosensitivity and oral ulcerations. Family history was also insignificant.

**Figure 1:**
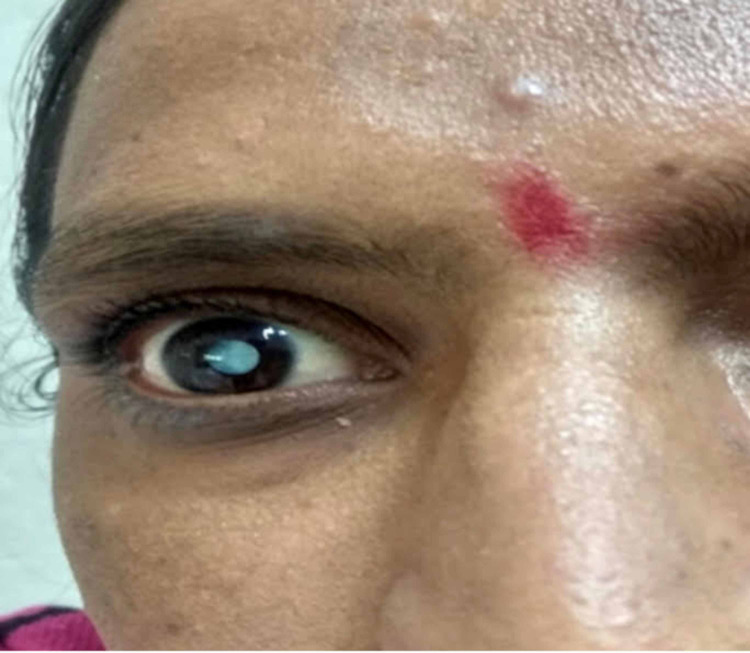
A mature cataract of right eye

On examination, her vitals were within normal range. Neurological examination revealed a positive Chvostek and Trousseau’s sign. There was no nystagmus, any abnormal movements or gait abnormality. Cardiovascular, respiratory, and abdominal examinations were unremarkable. Ophthalmological examination revealed a mature cataract in the right eye (Figure 1).

Serum calcium was 4.81 mg/dL (normal range 8.8-10.6 mg/dL), intact parathyroid hormone (PTH) 3.4 pg/mL (normal range 18.5 - 88.0 pg/mL), serum phosphate 7.90 mg/dL (normal range 2.5-4.5 mg/dL) with normal kidney functions. Lab results are shown in Table 1.

**Table 1:** Biochemical and hematological investigations

Parameters	Value	Normal Range
Serum calcium	4.81 mg/dL	8.8-10.6 mg/dL
Serum phosphorus	7.90 mg/dL	2.5-4.5 mg/dL
Serum sodium	137 mEq/L	135-145 mEq/L
Serum potassium	4.2 mEq/L	3.5-5.0 mEq/L
Serum 25 hydroxyl- vitamin D	56.6 ng/ml	30 - 100 ng/ml
Parathyroid hormone	3.4 pg/mL	18.5 – 88.0 pg/mL
Serum creatinine	0.68 mg/dL	0.51–1.2 mg/dL
Serum albumin	4.48 gm/dL	3.5-5.2 g/dL
Alkaline phosphatase	84 U/L	30–120 U/L
Hemoglobin	10.7gm/dL	13-17 gm/dl
Total leucocyte count	5700 /cumm	4000-10000/cumm
Fasting blood glucose	91 mg/dL	80-100 mg/dL
Thyroid-Stimulating Hormone	2.27 mU/L	0.4-4.2 mU/L

**Figure 2:**
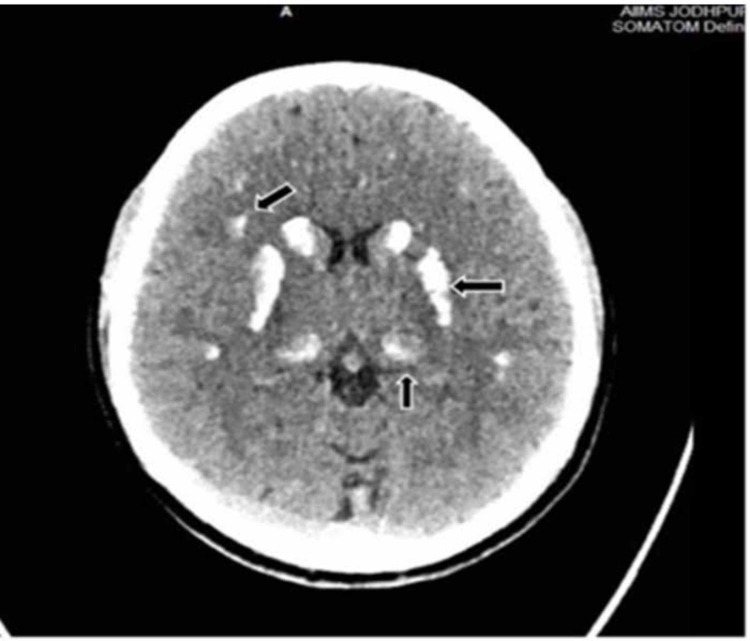
Non-contrast computed tomography Head showing large foci of parenchymal calcifications seen in bilateral putamen, thalami and grey-white matter junctions in bilateral frontal and parietal lobes. (Arrowed- Right frontal, left putamen and thalamus)

A non-contrast computed tomography scan of the head was suggestive of bilateral extensive intracranial calcification in the caudate, thalamus, lentiform nucleus and corona radiata (Figure 2).

Based on these biochemical laboratory reports and radiological findings, the diagnosis was made as primary hypoparathyroidism with cataract and extensive intracranial calcification. The patient was started on oral calcium and active vitamin D, following which she developed significant improvement in her symptoms. Serum calcium level raised to normal range and muscular spasms were subsided. The patient was referred to the department of ophthalmology for surgical management of right eye cataract.

## Discussion

Clinical presentation of hypoparathyroidism varies depending on the duration of hypocalcemia. Muscular cramps, tetany, paraesthesia, cardiac arrhythmias, altered mental status and seizures are usually acute manifestations. Cataract, basal ganglia calcifications, dilated cardiomyopathy, dementia and cerebellar dysfunction are chronic manifestations [4].

Cataract in young patients is usually secondary to eye trauma, uncontrolled diabetes, galactosemia, hypothyroidism, inborn error of copper metabolism and chronic hypocalcemia [5]. Cataract is a well-known complication of hypoparathyroidism. The progression of cataract is usually slow in patients with primary hypoparathyroidism while cataracts due to secondary hypoparathyroidism develop rapidly. Pathogenesis of cataract in hypoparathyroidism is proposed as membrane damage due to low calcium levels in the aqueous humour [6].

Extensive intracranial calcification is an occasional complication of hypoparathyroidism. Factors that predispose this brain calcification have not been completely elucidated. Prolonged hypocalcemia and hyperphosphatemia promote the deposition of calcium-phosphorus complexes in brain parenchymal tissue. Increased expression of osteogenesis-related molecules like osteonectin/osteopontin in the caudate nucleus and grey matter could favour metastatic calcification in hypoparathyroidism [7,8].

Auto-immune hypoparathyroidism and genetic causes are important etiologies of primary hypoparathyroidism [9]. However, anti-calcium sensing receptor (CaSR) antibodies and genetic testing for Glial Cell Missing-2 (GCM2) or CaSR genetic mutations could not be done due to non-availability of these testing facilities in our institute and economic constraints, thus these causes could not be excluded.

On review of the literature, we found various case reports of hypoparathyroidism with cataract or basal ganglia calcification. Goswami et al. [10] concluded in their study that 50% of patients of idiopathic hypoparathyroidism had basal ganglia calcification with cataract, but there are very few reports of cataract along with intracranial calcification in primary hypoparathyroidism (Table 2).

**Table 2:** Review of literature for cataract and brain calcification in hypoparathyroidism

Authors/ reference no	Patient profile	Duration of disease	Remarks
Xuan Liao et al. ^[3]^	37 years/male	11 years	Bilateral cataract with seizures, Brain imaging is not mentioned
Ramen C. Basak ^[8]^	40 years/male	Acute onset seizures	Bilateral calcification in thalamus, dentate nuclei, putamen and cerebellum
Khalil Ahmed et al.^ [11]^	20 years/female	3 years	Bilateral cataract with alopecia
Moushumi Lodh et al. ^[12]^	30 years/female	6 years	Basal ganglia calcification with seizures
Ko Harada et al. ^[13]^	70 years/male	Acute onset hemiparesis	Bilateral calcifications in basal ganglia, and cerebellum with acute cerebral infarct
Zhou YY et al. ^[14]^	62 years/male	10 years	Bilateral calcification in basal ganglia and cerebellum, presented with tetany and speech difficulty

In our patient, there was a right eye cataract and bilateral extensive intracranial calcification with a history of cataract surgery of the left eye two years back. If this patient was subjected to further evaluation of cataract at such a young age, perhaps she could have been prevented from getting cataract in the other eye.

## Conclusions

We would like to highlight that cataract in young patients is always a matter for further evaluation. Clinicians and ophthalmologists should be aware of hypoparathyroidism as a cause of bilateral cataract. Early diagnosis of primary hypoparathyroidism can save patients from many complications.
